# Complete response of hepatocellular carcinoma with right atrium and pulmonary metastases treated by combined treatments (a possible treatment effect of natural killer cell): A case report and literature review

**DOI:** 10.1097/MD.0000000000012866

**Published:** 2018-10-19

**Authors:** Dong Hyun Kim, Eunae Cho, Sung Bum Cho, Sung Kyu Choi, Sunmin Kim, Jieun Yu, Young-Il Koh, Da Woon Sim, Chung Hwan Jun

**Affiliations:** a Division of Gastroenterology; b Division of Allergy, Asthma, and Clinical Immunology, Department of Internal Medicine, Chonnam National University Hospital and Medical School, Gwangju, South Korea.

**Keywords:** anaphylaxis, antitumor activity, hepatocellular carcinoma, natural killer cell, neoplasm metastasis

## Abstract

**Rationale:**

Hepatocellular carcinomas (HCCs) with metastases to the right atrium (RA) and lungs are rare, with a poor prognosis. Furthermore, the treatment outcomes in patients with advanced HCCs remain unsatisfactory.

**Patient concerns:**

A 46-year-old man presented to our hospital for dyspnea on exertion and abdominal pain.

**Diagnoses:**

HCC and extra-hepatic metastases to the lung and RA.

**Interventions:**

Multidisciplinary treatment including radiotherapy (RT), transarterial chemoembolization (TACE), and sorafenib. During a follow-up evaluation computed tomography, he experienced a radio-contrast-induced anaphylaxis. After the event, treatment such as RT, TACE, and sorafenib were continued.

**Outcomes:**

His tumor burden decreased, finally leading to a complete response as per the modified Response Evaluation Criteria in Solid Tumors. The patient is still alive, 30 months after the episode. Subsequent blood tests showed increased natural killer (NK) cell activity, which was significantly higher than that seen in other age-matched HCC patients with an identical stage of the tumor, receiving sorafenib. This suggests that the increase in NK cells induced by anaphylaxis influenced the tumor burden.

**Lessons:**

We report here a rare case of long-term survival of an HCC patient with multiple metastases treated with multidisciplinary modalities, in which high NK cell activity was observed after a radio-contrast-induced anaphylactic reaction during follow-up investigations.

## 1 Introduction

Hepatocellular carcinoma (HCC) is the fifth most common cancer worldwide and the third leading cause of cancer-related death.^[1]^ A number of systems have been proposed to predict the prognosis for HCC. Union Internationale Contre Le Cancer (UICC) tumor-node-metastasis classification,^[2]^ Cancer of the Liver Italian Program (CLIP) score,^[3]^ and Barcelona Clinic Liver Cancer (BCLC) staging indicate the prognosis of HCC.^[4]^

HCC is typically diagnosed late in the course of the disease, and the median survival after diagnosis ranges approximately between 6 and 20 months.^[5]^ Vascular invasion and extrahepatic spread worsen the prognosis.^[6]^ Most current guidelines recommend sorafenib-based diverse treatment for advanced HCC.^[7,8]^ Although a randomized, double-blind, placebo-controlled trial, the Sorafenib Hepatocellular Carcinoma Assessment Randomized Protocol (SHARP), and an Asia-Pacific study demonstrated the efficacy and safety of sorafenib in increasing the period of survival by 2 to 3 months compared with the control group, complete response (CR) was not observed in these studies.^[9,10]^

Anaphylaxis is an immunoglobulin E (Ig E)-mediated hypersensitivity reaction. It is a critical allergic reaction, which is rapid in onset and can cause death. It is often accompanied by hypotension, gastrointestinal symptoms, respiratory symptoms, or skin symptoms.^[11]^

Mast cells and many of their products are known for their association with various conditions, such as asthma, allergy, and anaphylaxis. Recent studies have indicated that activation of the mast cells might lead to selective chemotaxis of the natural killer (NK) cells by CXCL8- and CXCR1-dependent mechanisms.^[12,13]^ NK cells were originally defined as effector lymphocytes of innate immunity, with constitutive cytolytic functions. Recent studies have revealed that NK cells can also contribute to adaptive immunity.^[14]^ NK cells are cytotoxic and are known to be effective for various types of tumor cells.^[15]^

Recently, Sun et al reported that NK cell dysfunction occurs in HCC, and NK cell-based anti-HCC treatment strategies alone or in combination with other therapies might be effective.^[16]^

We report here a rare case of long-term survival of an HCC patient with multiple metastases treated with multidisciplinary modalities, in which high NK cell activity was observed after a radio-contrast-induced anaphylactic reaction during follow-up investigations.

## 2 Case report

**Figure 1:**
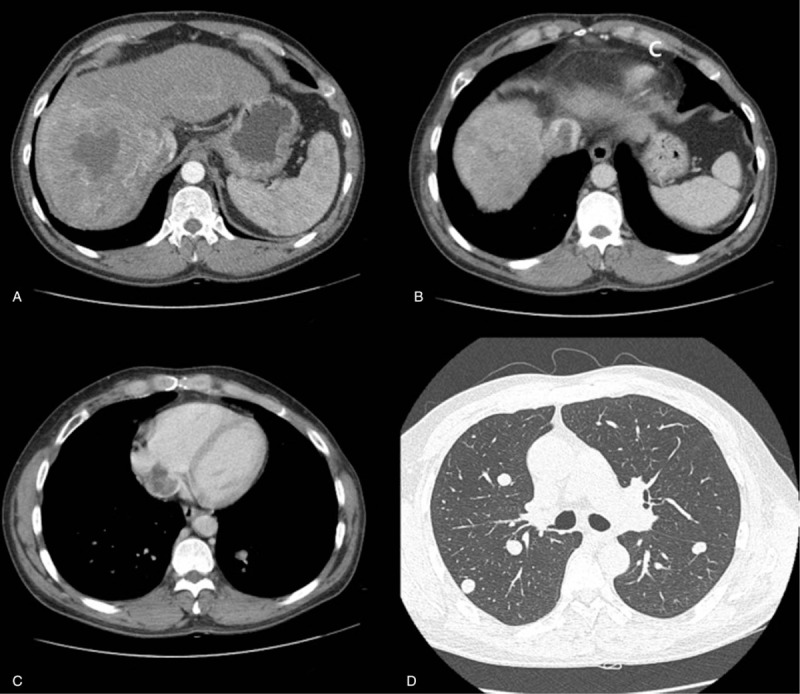
Initial abdominal computed tomography (CT) revealed a 12.6 × 12.2-cm-sized hepatocellular carcinoma with daughter nodules in the right hepatic lobe (A), tumor thrombosis in the suprahepatic inferior vena cava (B), and metastasis to the right atrium (C). Chest CT showed multiple well-defined nodules in both lungs, suggesting pulmonary metastasis (D).

In November 2015, a 46-year-old man presented to our hospital for dyspnea on exertion and abdominal pain, since a week. He had chronic hepatitis B-related liver cirrhosis, without any other disease. On admission, his performance score (Eastern Cooperative Oncology Group performance status) was 1. Initial laboratory investigations showed a total bilirubin level of 1.93 mg/dL, albumin of 3.9 g/dL, and prothrombin time international normalized ratio of 1.13. Shifting dullness or abdominal distension was not observed, and his mental state appeared normal. The cirrhosis was classified as Child-Pugh class A6. Initial computed tomography (CT) scan of the chest and abdomen demonstrated a 12.6 × 12.2 × 11.0 cm HCC with daughter nodules in the right hepatic lobe and tumoral thrombosis in the intrahepatic and suprahepatic inferior vena cava (IVC) and right atrium (RA), as well as multiple lung metastases (Fig. 1). Transthoracic echocardiography revealed a heterogeneous oscillating mass from the distal IVC to the RA. The initial alpha-fetoprotein (AFP) level was 33,989 ng/mL. The tumor conformed to the BCLC stage C, with a CLIP score of 3, American Joint Committee on Cancer TNM staging systems (AJCC TNM) stage IVB, and modified UICC stage IVB.

**Figure 2:**
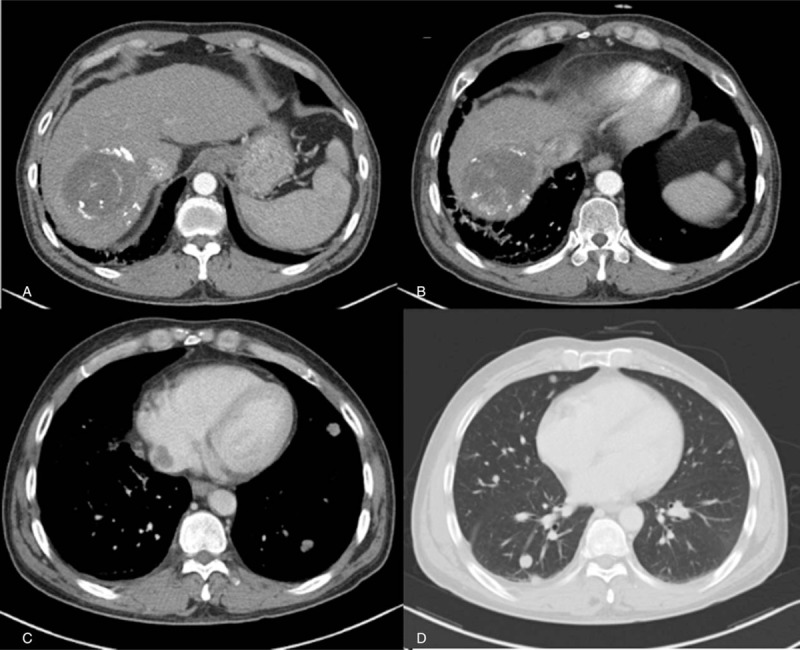
Follow-up abdominal computed tomography (3 months after diagnosis) (CT) revealed about 8.5 × 7.0-cm-sized decreased size of necrotic mass of hepatocellular carcinoma in right hepatic lobe (A), mildly decreased tumoral thrombosis in suprahepatic inferior vena cava (B) and decreased size of metastasis of right atrium (C). Chest CT showed multiple newly detected nodules in both lungs, suggesting pulmonary metastasis (D).

**Figure 3:**
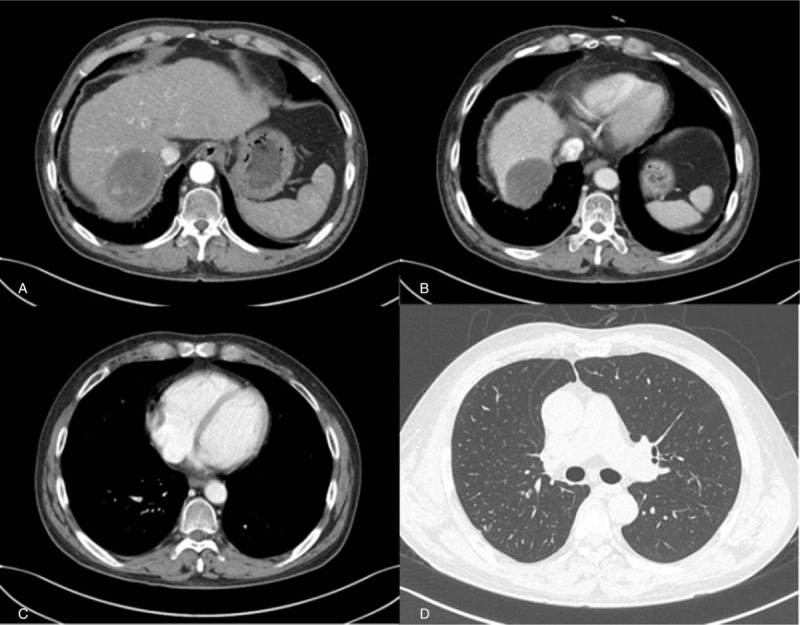
Two years later, follow-up abdominal computed tomography (CT) revealed a 6.4 × 5.2-cm-sized and significantly reduced necrotic hepatocellular carcinoma in the right hepatic lobe, without tumoral enhancement (A). Nearly resolved tumoral thrombosis in the suprahepatic inferior vena cava (B) and complete resolution of the right atrium metastasis was seen (C). No detectable pulmonary nodules in both lungs on Chest CT (D).

Immediately after the diagnosis, he was treated with entecavir 0.5 mg daily, and sorafenib 400 mg twice daily (Nexavar, Bayer, Germany, Leverkusen), which was reduced to 400 mg a day because of a grade III hand–foot skin reaction (HFSR) after 1 month. Ten days after the diagnosis, palliative radiation therapy (daily 250 cGy, 14 times, total 3500 cGy) to the hepatic mass, IVC, and RA metastasis was initiated. Three weeks later, on December 8, 2015, the first transarterial chemoembolization (TACE) was performed with Lipiodol 6 cc/Adriamycin 20 mg. After 6 weeks of treatment, on December 31, 2015, a follow-up CT was conducted to check the tumor response. The contrast medium used was iomeprol (iomeron; Bracco Imaging Korea, Ltd., Korea, Seoul), and it was the first time he had received the agent. He had no history of adverse effects from other contrast agents used previously. However, the patient complained of dyspnea and developed loss of consciousness, immediately after injection of the contrast medium iomeprol. The systolic blood pressure was 50 mm Hg, whereas the diastolic BP could not be detected. After injecting 1 mg of epinephrine, 4 mg of peniramine, 5 mg of dexamethasone, and loading 2 LL of normal saline, he gradually recovered from the anaphylactic shock. ImmunoCAP tryptase measured 1 hour after the event was increased to 19.8 μg/L (normal range: 0–11.5 μg/L). Follow-up CT revealed that the tumor mass was reduced to 9.1 × 7.5 × 7.3 cm (from 12.6 × 12.2 × 11.0 cm) in the right hepatic lobe. Necrotic changes were observed in the mass, and the extent of tumor thrombosis in the intrahepatic and suprahepatic IVC and the RA had decreased. However, multiple newly nodules in both lung (pulmonary metastasis) were detected (Fig. 2). Based on the modified Response Evaluation Criteria in Solid Tumors criteria,^[17]^ overall partial response (PR) was noted. Sorafenib administration was continued and the second TACE was performed in February 2016. After 3 weeks of treatment with second TACE, a follow-up CT (February 26, 2016) revealed more regression of intrahepatic mass, IVC, and RA. However, both pulmonary metastases were aggravated. Fourteen months of sorafenib treatment, 2 sessions of TACE, and a follow-up CT (June 24, 2016) revealed the tumor burden had decreased considerably including both pulmonary metastases, with extensive necrotic areas and no tumoral enhancement, and sustained normalization of the alpha-fetoprotein level was noted (Fig. 3).

**Figure 4:**
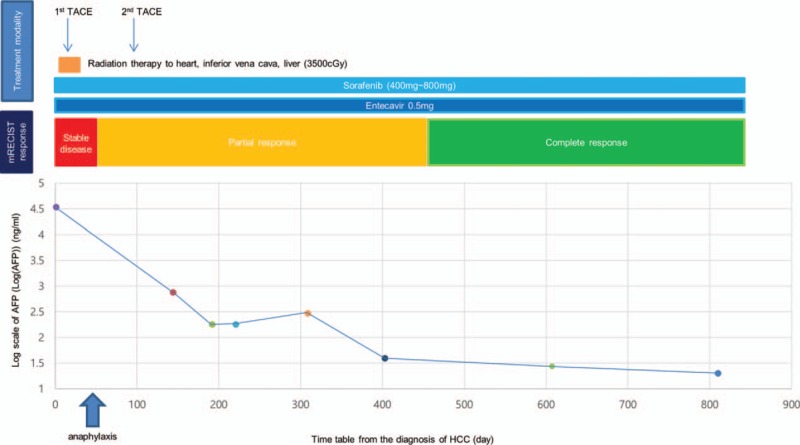
Clinical course of the patient. AFP = alpha-fetoprotein, cGy = centigray, HCC = hepatocellular carcinoma, mRECIST = modified Response Evaluation Criteria in Solid Tumors, TACE = transarterial chemoembolization.

The NK cell activity of our patient was checked, and the value of NK cell activity was significantly higher than normal range (CD56: 54.1%, normal range: 6%–37%). Thirty months after the initial diagnosis, the patient is still alive with no clinical or radiological evidence of tumor recurrence (Fig. 4).

## 3 Discussion

The prognosis of HCC with multiple metastases is very poor. The median survival of these patients without effective treatment has been reported to be as short as 2 to 3 months.^[18]^ According to the SHARP trial and an Asia-Pacific study, only 2% to 3.3% cases had PR and CR was not seen.^[9,10]^ To our knowledge, only 18 cases of stage IV HCC receiving sorafenib or multimodality treatments have achieved CR so far.^[19–33]^ Therefore, we have listed 19 published cases of stage IV HCC in the sequential order of publication, including our case (Table 1). Of these, 18 patients (94.7%) were males. The mean age was 64.3 (range, 46–78) years. The cancer stage as per AJCC TNM staging system was IVA in 21.1% and IVB in 78.9% patients, respectively. The median time to CR was 6 months (1–48 months). Eighteen patients (94.7%) were treated with sorafenib. TACE (63.2%), surgical resection (47.4%), radiofrequency ablation (21.1%), RT (15.8%), and systemic chemotherapy (10.5%) were used as adjuncts. HFSR was seen in 11 patients (57.9%); of these 6 patients were in grade 3 and 5 patients were in grade 2. Diarrhea (5 patients, 26.3%) was the second most common adverse effect. To summarize these case reports, most patients who received sorafenib were males and a significant number of patients suffered HFSR. This is similar to our case.

**Table 1:**
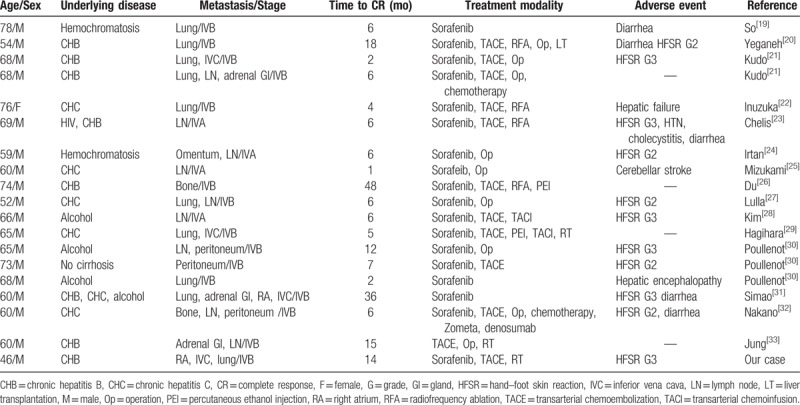
Review of 19 CR cases of stage IV HCC in English literature.

Some studies have shown that multimodal therapy is effective in advanced HCC with RA metastasis.^[34]^ Sorafenib enhances the radiosensitivity of human HCC cell lines by selectively inhibiting the radiation-induced activation of the vascular endothelial growth factor receptor 2 and extracellular signal-regulated kinase pathways, thereby promoting radiation-induced apoptosis.^[35]^ TACE has been reported to be an effective treatment modality for advanced HCC, and there are reports that combined treatment with TACE and sorafenib has better outcomes than TACE alone.^[36]^ There was also a case report of advanced HCC cured by TACE, RT, and surgery without sorafenib.^[33]^ Similar to other reports, concomitant therapy with sorafenib, RT, and TACE might have had a synergistic effect on the patient's prognosis in our case.

**Figure 5:**
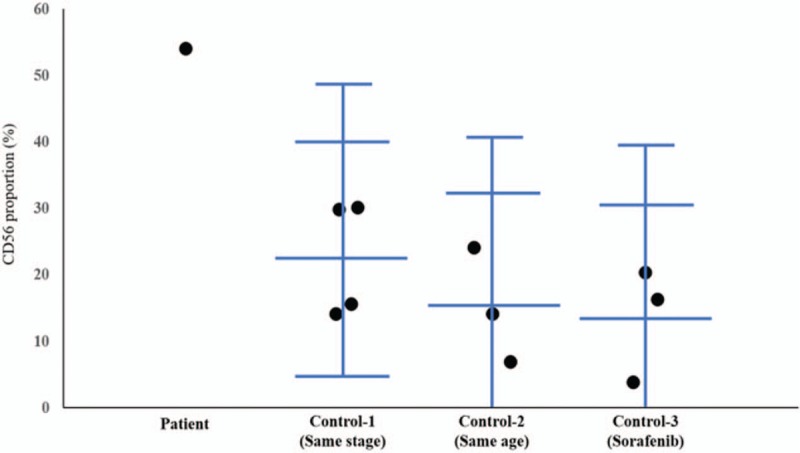
Comparison of the natural killer cell activity (CD56) in the patient and in the 3 control groups. The longest transverse bar indicates the average value in each group, the second transverse vertical bar indicates the average value ±2 SD of each group and the shortest transverse bar indicates the average value +3 SD of each group.

A point to note in our case is that the patient's tumor burden rapidly decreased and he eventually achieved CR after the anaphylaxis event. We assume a possible synergistic effect of the anaphylaxis and multimodal treatment in tumoral response. The mechanism might be explained by the elevation of serum tryptase level during anaphylaxis, which indicates activation of the mast cells. Based on the result of recent studies, the activation of mast cells might lead to a selective chemotaxis of the NK cells, which might play a role in anticancer effect. Another study showed that the NK cells led to long-term survival in the livers of nude mice, and inhibited lung metastasis of HCC in vivo.^[37]^ Another study revealed that the quantities and activities of NK cells were significantly lower in HCC patients compared with healthy individuals.^[38]^ In addition, among patients with HCC, a high NK cell number and activity are associated with early stages and improved patient survival.^[39,40]^ Based on the above results, NK cell activity of the patient was checked and to our surprise, subsequent blood tests showed a markedly increased NK cell (CD56) activity compared with the normal levels. Recently, some studies reported that sorafenib might influence the activity of NK cells and play a role in the anticancer effect by modulating macrophages and NK cells.^[41,42]^ In our case, the patient was continuously on sorafenib; it might be useful to check the NK cell activity in patients with advanced HCC receiving sorafenib, for comparison. To compare the NK cell activity of the patient with that of other HCC patients, other patients diagnosed with HCC were considered as a control group and their NK cell activity was measured. Control groups were divided into 3 categories: HCC patients with similar age (n = 3), HCC patients with the same stage of tumor (stage IV) (n = 4), and HCC patients receiving sorafenib (n = 3). The NK cell activity in the HCC patients of similar age was 15.07 ± 8.63%, in HCC patients with the same stage of tumor (stage IV) was 22.48 ± 8.77%, and in HCC patients receiving sorafenib was 13.53 ± 8.59%. The NK cell activity of our patient was significantly higher than that seen in patients of the control groups (CD56: 54.1%, normal range: 6%–37%) (Fig. 5). It is possible that the baseline NK cell level of our patient might have been high, the anaphylaxis might have been an accident, and the high proportion of NK might not be related to the anaphylaxis. However, it is known that NK cell activity is low in HCC patients; after the adjustment for the age of patients, the activity of NK cells is lower in the patients in advanced stage HCC.^[43–45]^ The activity of NK cells in our patient was significantly higher than that in patients with the same stage of disease, of a similar age, and receiving sorafenib. Based on several other study findings,^[39,40]^ we assume that our patient would have had low levels and activities of NK cells when he was diagnosed with HCC with multiple metastases. Therefore, it can be assumed that the activation of mast cells induced by anaphylaxis might have influenced the activation of NK cells in our patient. However, the exact mechanism due to which NK cell levels remained high for a long time after the anaphylaxis is not known.

There are some limitations in our case. First, we did not check the baseline NK cell level of the patient; hence, we could not compare the baseline NK cell level with the NK cell level after anaphylaxis. Second, the mechanism by which the mast cell activates the NK cell is not understood. Nevertheless, this case report could help develop newer therapies that can improve the prognosis for patients with end-stage liver cancer. In addition, the effect of mast cell activation on NK cells might be worth investigating in the development of therapeutic agents for other cancer patients. Further studies are needed to determine whether the activation of mast cells can activate NK cells and produce anticancer effects.

## Author contributions

DHK, EC, SBC, SKC, JY, and CHJ collected patient's data and contributed to manuscript writing. DHK, YIK, SMK, and DWS analyzed data. DWS and CHJ designed research studies and wrote the manuscript.

**Conceptualization:** Dong Hyun Kim, Eunae Cho.

**Data curation:** Dong Hyun Kim, Eunae Cho, Sung Bum Cho, Sung Kyu Choi, Jieun Yu, Chung Hwan Jun.

**Formal analysis:** Sunmin Kim, Young-Il Koh, Da Woon Sim.

**Investigation:** Dong Hyun Kim, Sunmin Kim.

**Methodology:** Da Woon Sim.

**Visualization:** Sunmin Kim.

**Writing – original draft:** Dong Hyun Kim, Eunae Cho, Sung Bum Cho, Jieun Yu, Chung Hwan Jun.

**Writing – review and editing:** Dong Hyun Kim, Sung Bum Cho, Sung Kyu Choi, Young-Il Koh, Da Woon Sim, Chung Hwan Jun.

Dong Hyun Kim orcid: 0000-0001-5778-1264.
